# Tongue pressure in patients with post-stroke dysphagia

**DOI:** 10.3389/fphys.2026.1889535

**Published:** 2026-10-27

**Authors:** Qingjia Cui, Qian Zhang, Weiwei Jia, Haiying Wang, Lei Zhang, Xin Dai

**Affiliations:** Rehabilitation Centre of Speech Therapy, Department of Otolaryngology, Beijing Rehabilitation Hospital, Capital Medical University, Beijing, China

**Keywords:** aspiration, dysphagia, five-point measurement, post-stroke, tongue pressure

## Abstract

**Introduction:**

Tongue pressure is essential for oropharyngeal swallowing, but regional normative and pathological values remain unclear, as do its links to swallowing and aspiration in post-stroke dysphagia (PSD). This study assessed maximal tongue pressure (Pmax) at five sites (anterior, middle, posterior, left anterior, right anterior) in healthy individuals and PSD patients, and examined its correlations with swallowing physiology and aspiration in PSD.

**Methods:**

We enrolled 70 post-stroke dysphagia patients (DSG), 70 post-stroke non-dysphagia patients (NDSG), and 70 healthy controls (CG). Pmax was compared among groups. Within DSG, correlations between Pmax and VFSS/FEES parameters and aspiration were analyzed.

**Results:**

In all three groups, posterior Pmax was highest, right anterior lowest (*P* < 0.001). Pmax ranked CG > NDSG > DSG at all sites (all *P* < 0.001). In DSG, posterior Pmax was associated with cricopharyngeal opening (*P* = 0.030) and pharyngeal residue (*P* = 0.014) on VFSS, but not with any FEES parameter. All five Pmax values negatively correlated with oral transit time (all *P* < 0.001), strongest in left/right anterior. Soft palate elevation time weakly negatively correlated with left anterior, middle, and right anterior Pmax (*r* = -0.252 to -0.248, *P* < 0.05). Hyoid movement time weakly positively correlated with left anterior, anterior, and right anterior Pmax (*r* = 0.251-0.340, *P* < 0.05). Pharyngeal phase initiation time negatively correlated with middle Pmax (*r* = -0.286, *P* = 0.016) and posterior Pmax (*r* = -0.313, *P* = 0.008). No Pmax site predicted aspiration on VFSS/FEES. PSD, NDSG, and CG all showed highest posterior and lowest right anterior pressure.

**Discussion:**

In PSD, anterior pressure aids oral transport, posterior pressure relates to pharyngeal function. Tongue pressure poorly predicts aspiration.

## Introduction

Post-stroke dysphagia is one of the most common and serious complications of stroke. Epidemiological data indicate that the incidence of dysphagia in acute stroke patients ranges from 30% to 78% (Martino et al., 2005). Its high incidence significantly affects patients’ rehabilitation progress and quality of life while also imposing a substantial burden on healthcare systems (Rofes et al., 2013; Al Rjoob et al., 2022; Karisik et al., 2024).

During oropharyngeal swallowing, the tongue performs multiple key physiological functions, including bolus formation and propulsion, triggering of the swallowing reflex, sealing of the posterior oral cavity, and prevention of food regurgitation. The pressure generated when the tongue contacts the hard palate is defined as tongue pressure. During forceful swallowing, increased tongue pressure accelerates pharyngeal pressure generation and promotes pressure wave propagation into the pharyngeal cavity, thereby facilitating smooth bolus passage into the esophagus. Reduced tongue pressure is closely associated with dysphagia, primarily due to decreased tongue-palate contact pressure resulting from long-term functional decline of the tongue muscles, which in turn affects swallowing efficiency and safety (Konaka et al., 2010; Shimizu et al., 2021). Therefore, tongue pressure assessment has significant clinical value for evaluating swallowing function.

Post-stroke tongue dysfunction particularly affects the oropharyngeal phase of swallowing. Reduced tongue pressure not only decreases swallowing efficiency and prolongs the oral phase but also impairs bolus transit (Lee et al., 2016). When tongue propulsion capacity is compromised, tongue pressure decreases accordingly, potentially allowing liquid or food to flow into the pharynx before airway protection mechanisms are activated, thereby increasing the risk of aspiration. Tongue pressure is significantly lower in individuals with aspiration than in those without aspiration (Nakamori et al., 2021), and patients with dysphagia generally have lower tongue pressure than non-dysphagic individuals and healthy controls (Galek et al., 2022; Van der Straeten et al., 2025). Some researchers have suggested that tongue pressure may also serve as a sensitive indicator for predicting the risk of post-stroke pneumonia (Szabó et al., 2023; Ohashi et al., 2025).

Tongue pressure measurement, as a rapid, objective, and non-invasive method for assessing tongue muscle strength, has been widely adopted for swallowing function evaluation. Traditional tongue pressure measurement tools mostly focus on global tongue pressure and are limited in their ability to fully capture the functional status of individual tongue regions. Therefore, this study employed a T-type five-point tongue pressure measurement probe to measure maximal tongue pressure at the left anterior, anterior, middle, posterior, and right anterior regions in the post-stroke dysphagia group (DSG), non-dysphagic post-stroke group (NDSG), and control group (CG). Through three-group comparative analysis, we aimed to characterize post-stroke changes in regional tongue muscle strength. Furthermore, we analyzed the correlations between tongue pressure measurements in the DSG and various physiological parameters and aspiration derived from videofluoroscopic swallowing study (VFSS) and fiberoptic endoscopic evaluation of swallowing (FEES), with the goal of providing objective references for comprehensive dysphagia assessment and aspiration screening, and offering theoretical guidance for subsequent rehabilitation interventions.

## Materials and methods

### Patients and participant selection

This single-center case-control study retrospectively enrolled 70 patients with confirmed post-stroke dysphagia from the Speech Rehabilitation Center of our hospital between January 2025 and January 2026. The DSG comprised 55 males and 15 females, with a mean age of 59.6 ± 18.0 years.

Inclusion criteria: (1) meeting Chinese diagnostic criteria for cerebral infarction or hemorrhage (2007) (Expert Consensus Group on Rehabilitation Assessment and Treatment of Dysphagia in China, 2017); (2) dysphagia confirmed by videofluoroscopic swallowing study (VFSS); (3) disease duration ≤ 2 years; (4) written informed consent. Exclusion criteria: (1) severe cognitive or communication disorders; (2) primary gastrointestinal disease or inability to cooperate; (3) serious complications (e.g., respiratory, heart, liver, or renal failure; severe pulmonary infection); (4) head/neck diseases affecting swallowing; (5) prior oral/pharyngeal surgery.

The NDSG consisted of 70 post-stroke patients without dysphagia, matched for demographic characteristics. The absence of dysphagia in the NDSG was confirmed by a trained speech-language pathologist using a standardized clinical swallowing assessment, including the Eating Assessment Tool (EAT-10) questionnaire and the 30-mL water swallow test; patients with abnormal screening results or any complaint of swallowing difficulty underwent VFSS and were excluded from the NDSG. Seventy healthy individuals were enrolled as the control group. No significant differences in demographic or clinical characteristics were observed among the three groups (**Table 1**).

**Table 1:** Demographic and clinical characteristics of the three groups (N, %).

Items	DSG (n=70)	NDSG (n=70)	CG (n=70)
Sex			
Male	55 (78.6)	50 (71.4)	53 (75.7)
Female	15 (21.4)	20 (28.6)	17 (24.3)
Age (years)	59.6 ± 18.0 (14-85)	56.9 ± 14.6 (20-83)	57.8 ± 19.6 (16-80)
Dysphagia	70 (100)	0	0
Aspiration			
VFSS	24 (34.3)	0	–
FEES	45 (64.3)	0	–
StrokeSubtypes			
ATBI	16 (22.9)	18 (25.7)	–
CEI	17 (24.3)	15 (21.4)	–
LI	12 (17.1)	14 (20.0)	–
ICH	13 (18.6)	11 (15.7)	–
Others	12 (17.1)	12 (17.1)	–

DSG, post-stroke dysphagia group; NDSG, post-stroke non-dysphagia group; CG, control group; VFSS, videofluoroscopic swallowing study; FEES, fiberoptic endoscopic evaluation of swallowing; ATBI, atherothrombotic infarction; CEI, cardioembolic infarction; LI, lacunar infarction; ICH, intracerebral hemorrhage.

### Ethics approval

This study was approved by the Institutional Review Board of Beijing Rehabilitation Hospital, Capital Medical University (No. 2023-15). Written informed consent was obtained from all participants.

### Maximum tongue pressure

Maximum tongue pressure (Pmax) and tongue endurance were measured using a swallowing neuromuscular electrical stimulator (Longest, model LGC-2352DE) (**Figure 1A**). A T-shaped five-point tongue pressure sensor (**Figure 1B**), comprising force-sensitive resistors, a sensing pad, and a recording device, simultaneously measured pressure at five oral regions (anterior, middle, posterior, left anterior, and right anterior). The five sites were selected to cover the anterior–posterior and left–right axes of the tongue–palate contact area, based on previous multi-point tongue pressure studies and the functional specialization of the anterior, middle, and posterior tongue (Hirota et al., 2010; Rosen et al., 2017; Bahia and Lowell, 2024). This multi-site approach was intended to improve both reliability and sensitivity compared with single-site measurement.

**Figure 1:**
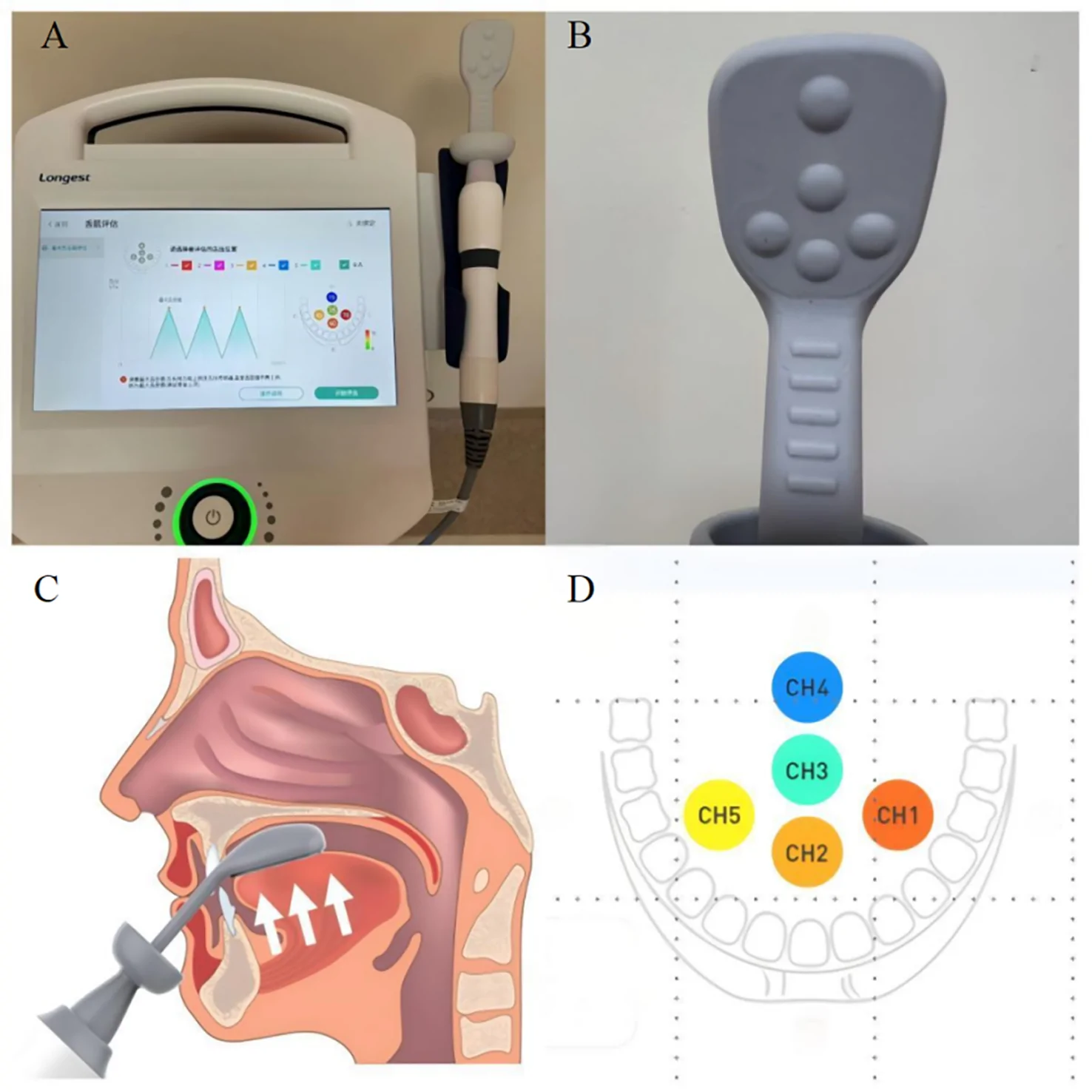
Maximum tongue pressure. **(A)** Measurement device (swallowing neuromuscular electrical stimulator). **(B)** T-shaped five sites tongue pressure sensor. **(C)** Sensor compression during tongue elevation against the hard palate. **(D)** Maximal pressure data from channels CH1–CH5.

Before each measurement session, the device was calibrated according to the manufacturer’s instructions. The T-shaped sensor pad was positioned on the hard palate so that the five sensing elements contacted the midline (anterior, middle, posterior) and bilateral anterior regions. Participants sat upright with the Frankfort horizontal plane parallel to the floor and were instructed to elevate the tongue maximally against the hard palate. The validity and test-retest reliability of this sensor still require further validation, as acknowledged in the Limitations. The sensor was firmly attached to the participant’s tongue. During active tongue elevation against the hard palate, sensor compression (**Figure 1C**) yielded maximal pressure data for channels CH1-CH5 (**Figure 1D**). The measurement was repeated three times, and the mean value for each site was calculated as Pmax (left anterior, anterior, middle, posterior, and right anterior).

### Videofluoroscopic swallowing study

VFSS was performed using an OPERA digital multifunctional gastrointestinal imaging system (GMM Group), as in our previous study (Cui et al., 2023). Patients were seated upright in a lateral viewing position, and the field of view included the oral cavity, pharynx, larynx, and upper esophageal sphincter. Each patient was seated upright and swallowed a 5-mL bolus of medium-consistency (nectar-like) paste prepared by mixing iohexol with a commercially available xanthan-gum-based thickener according to the manufacturer’s instructions. The bolus was recorded in the anteroposterior and lateral views as it passed through the oral cavity, pharynx, and esophagus. In this study, each patient performed one swallow. Two independent blinded clinicians (a speech-language pathologist and a dysphagia specialist) evaluated the recordings; disagreements were resolved by discussion. Images were acquired by an experienced radiologist and a rehabilitation physician.

Six physiological parameters were graded (oral motor function, swallowing reflex, soft palate lift, cricopharyngeal muscle opening, glottic closure, pharyngeal residue) following previous methods (Cui et al., 2023). Quantitative VFSS analysis included kinematic parameters (anterior/superior hyoid displacement) and temporal parameters [oral transit time, soft palate elevation time, hyoid pause time, hyoid movement time (ms), pharyngeal swallow initiation time].

### Fiberoptic endoscopic evaluation of swallowing

FEES was performed using an electronic nasolaryngoscope (ATMOS SCOPE) by an experienced speech-language pathologist, following a standardized protocol. With the patient seated upright, the endoscope was passed transnasally, and the pharynx and larynx were examined at rest. Anatomical structures and function at rest were inspected first (velopharyngeal closure, pharyngeal wall contraction, base-of-tongue bulk, epiglottic inversion, laryngeal elevation, and vocal fold mobility). Each patient received a 5-mL bolus of plain yogurt mixed with one drop of green edible food coloring per 5 mL. The bolus was administered by spoon, and at least two swallows were observed. Residue location and amount after swallowing were recorded. Eight FEES components were evaluated: swallowing movements, swallowing reaction, larynx lift function, lingual surface closure, glottic closure, vallecular residue, pyriform sinus residue, and cough reflex. Because vallecular and pyriform sinus residue showed identical grade distributions in this cohort, they are presented together in **Table 2** as “Vallecular/Pyriform sinus residue”. All FEES recordings were scored independently by two blinded clinicians (a speech-language pathologist and a dysphagia specialist), and disagreements were resolved by discussion.

**Table 2:** Maximum tongue pressure (kPa) corresponding to different FEES physiological components in DSG.

FEES item	Grade	N	Left anterior	Anterior	Middle	Posterior	Right anterior
Swallowing movements	Normal	66	23.0 (3.2, 39.5)	15.0 (0.0, 34.2)	19.5 (8.0, 34.8)	30.0 (12.5, 49.0)	12.0 (0.0, 27.0)
	None	4	13.5 (8.0, 20.5)	11.0 (6.0, 17.2)	25.5 (18.2, 33.0)	36.5 (25.2, 42.8)	14.5 (9.2, 19.5)
	*H*		0.208	0.079	0.244	0.004	0.079
	*P*		0.648	0.778	0.621	0.950	0.778
Swallowing reaction	Normal	5	2.0 (0.0, 48.0)	16.0 (3.0, 28.0)	22.0 (12.0, 32.0)	28.0 (23.0, 63.0)	6.0 (3.0, 21.0)
	Delayed	65	23.0 (4.0, 38.0)	15.0 (0.0, 32.0)	21.0 (8.0, 35.0)	31.0 (12.0, 47.0)	12.0 (0.0, 27.0)
	*H*		0.603	0.005	0.095	0.188	0.011
	*P*		0.438	0.945	0.758	0.665	0.917
Larynx lift function	Normal	5	18.0 (3.0, 48.0)	17.0 (16.0, 28.0)	33.0 (32.0, 37.0)	39.0 (25.0, 63.0)	14.0 (3.0, 21.0)
	Impaired	33	22.0 (4.0, 32.0)	12.0 (0.0, 15.0)	16.0 (8.0, 28.0)	30.0 (12.0, 41.0)	12.0 (0.0, 20.0)
	Severe impaired	32	26.0 (3.8, 41.5)	19.5 (0.0, 41.2)	22.5 (9.2, 39.0)	32.0 (13.5, 51.8)	15.5 (0.0, 29.5)
	*H*		0.166	2.037	2.58	1.791	1.073
	*P*		0.920	0.361	0.275	0.408	0.585
Lingual surface closure	<10%	24	22.5 (8.2, 34.2)	13.0 (4.8, 19.8)	18.5 (10.2, 32.2)	29.5 (18.2, 46.0)	12.0 (3.0, 20.2)
	10–50%	16	19.0 (3.8, 30.2)	15.0 (0.0, 36.2)	24.5 (10.0, 38.8)	32.5 (14.8, 42.0)	12.0 (0.0, 30.2)
	>50%	30	26.0 (2.5, 40.8)	16.5 (0.8, 39.2)	20.0 (7.8, 34.8)	32.5 (8.8, 50.5)	13.5 (0.0, 28.5)
	*H*		0.047	0.402	0.208	0.034	0.022
	*P*		0.977	0.818	0.901	0.983	0.989
Glottic closure	<10%	24	25.0 (1.0, 32.0)	12.0 (10.0, 15.0)	22.0 (13.0, 28.0)	36.0 (30.0, 49.0)	12.0 (3.0, 16.0)
	10–50%	16	13.0 (3.0, 39.2)	11.0 (0.0, 38.8)	15.5 (4.0, 37.8)	28.5 (11.2, 42.8)	6.5 (0.0, 30.5)
	>50%	30	20.0 (11.5, 40.5)	18.0 (12.0, 34.5)	21.0 (12.0, 34.5)	29.0 (20.0, 48.0)	20.0 (13.0, 26.0)
	*H*		0.773	1.791	0.934	2.01	2.251
	*P*		0.679	0.408	0.627	0.366	0.324
Vallecular/Pyriform sinus residue	<10%	24	22.5 (8.2, 34.2)	13.0 (4.8, 19.8)	18.5 (10.2, 32.2)	29.5 (18.2, 46.0)	12.0 (3.0, 20.2)
	10–50%	16	19.0 (3.8, 30.2)	15.0 (0.0, 36.2)	24.5 (10.0, 38.8)	32.5 (14.8, 42.0)	12.0 (0.0, 30.2)
	>50%	30	26.0 (2.5, 40.8)	16.5 (0.8, 39.2)	20.0 (7.8, 34.8)	32.5 (8.8, 50.5)	13.5 (0.0, 28.5)
	H		0.047	0.402	0.208	0.034	0.022
	P		0.977	0.818	0.901	0.983	0.989
Cough reflex	Normal	22	30.5 (4.5, 51.2)	15.5 (10.2, 46.8)	21.5 (13.0, 45.0)	36.0 (20.0, 65.0)	13.5 (3.0, 40.0)
	Decreased	24	20.0 (1.5, 34.0)	12.0 (0.0, 32.0)	13.0 (5.5, 31.5)	29.0 (11.5, 45.0)	6.0 (0.0, 26.0)
	None	21	18.0 (5.0, 27.0)	14.0 (0.0, 18.0)	21.0 (12.0, 33.0)	29.0 (12.0, 42.0)	14.0 (7.0, 21.0)
	H		2.193	2.555	1.613	3.146	0.949
	P		0.334	0.279	0.446	0.207	0.622

FEES, fiberoptic endoscopic evaluation of swallowing.

### Penetration-Aspiration Scale

Penetration-Aspiration Scale (PAS) scores (8 levels, ≥6 = aspiration) were assigned to DSG patients from VFSS and FEES recordings (Borders and Brates, 2020). Level 1: No airway entry; Level 2: Entry into laryngeal vestibule (above glottis), cleared; Level 3: Entry into vestibule, not cleared; Level 4: Reaches glottis, cleared; Level 5: Reaches glottis, not cleared; Level 6: Passes below glottis, cleared; Level 7: Passes below glottis, not cleared despite effort; Level 8: Passes below glottis, no effort to clear. Passes below glottis, no effort to clear.

When applying the PAS to FEES recordings, the deepest extent of visible airway invasion immediately before and after each swallow was graded according to the same criteria, because white-out may obscure the pharyngeal phase; when airway invasion could not be visualized during the entire swallowing event, the swallow was classified on the basis of post-swallow images. All PAS scores were assigned independently by two blinded clinicians, and disagreements were resolved by discussion.

### Statistical analysis

Statistical analyses were performed as follows: (1) age and clinical scores were compared among the three groups using *one-way ANOVA* with *Bonferroni post hoc* tests; (2) within each group, differences in Pmax among the five tongue sites were analyzed using the Friedman test, followed by Wilcoxon signed-rank tests with Bonferroni correction for multiple comparisons; (3) between-group differences in Pmax at each site were analyzed using *one-way ANOVA*; (4) differences in Pmax across the grades of each VFSS or FEES physiological component were analyzed using the *Kruskal-Wallis* test; (5) associations between Pmax and the kinematic and temporal VFSS parameters were analyzed using *Pearson’s* correlation; and (6) binary logistic regression was used to analyze the predictive value of the five Pmax variables for aspiration (PAS ≥ 6) on VFSS and FEES, respectively. *P* < 0.05 was considered statistically significant.

## Results

### Maximum tongue pressure in the DSG, NDSG, and CG

Maximum tongue pressure for the three groups are shown in **Table 3**. Within the DSG, significant differences were found among the five sites: posterior Pmax was significantly higher, and right anterior Pmax significantly lower, than the other four sites (all *P<*0.001). Similar intra-group patterns were observed in the NDSG and CG. Between-group comparisons revealed significant differences at all five sites, with Pmax highest in the CG, followed by the NDSG, and lowest in the DSG (all *P<*0.001).

**Table 3:** Maximum tongue pressure (kPa) at five sites in the DSG, NDSG, and CG.

Group	Left anterior	Anterior	Middle	Posterior	Right anterior	*Friedman χ² (df = 4)*	*P*
DSG	24.31 ± 23.58	21.15 ± 23.90	24.25 ± 21.54	32.64 ± 22.61	19.43 ± 23.61	58.442	<0.001
NDSG	43.78 ± 25.83	41.81 ± 25.83	49.82 ± 23.09	52.76 ± 26.87	40.31 ± 28.05	17.197	0.002
CG	65.28 ± 12.32	64.56 ± 14.72	69.77 ± 15.86	74.94 ± 8.95	61.64 ± 15.10	66.698	<0.001
*F*	91.633	97.205	125.090	102.263	84.994		
*P*	<0.001	<0.001	<0.001	<0.001	<0.001		

DSG, post-stroke dysphagia group, NDSG, non-dysphagia post-stroke group, CG, control group.

### Correlation between maximum tongue pressure and VFSS or FEES physiological components in the DSG

As shown in **Table 4**, posterior Pmax was significantly associated with cricopharyngeal opening (*H* = 3.700, *P=*0.030) and with residue in the pyriform sinuses and valleculae (*H* = 3.811, *P* = 0.014). Higher posterior Pmax predicted better cricopharyngeal opening and less pharyngeal residue. No other significant associations were observed between Pmax at any site and VFSS physiological components (all *P*>0.05, **Table 4**).

**Table 4:** Maximum tongue pressure (kPa) corresponding to different VFSS physiological components in the DSG.

VFSS item	Grade	N	Left anterior	Anterior	Middle	Posterior	Right anterior
Oral motor	Normal	3	23.0 (16.0, 25.0)	12.0 (11.5, 13.5)	13.0 (12.0, 20.5)	29.0 (26.5, 35.0)	12.0 (12.0, 19.5)
	Impaired	25	17.0 (4.0, 32.0)	12.0 (1.5, 25.0)	21.0 (12.0, 34.0)	34.0 (23.0, 45.0)	14.0 (3.0, 25.0)
	Severe impaired	42	11.0 (2.0, 28.5)	5.5 (0, 17.8)	17.5 (5.8, 29.0)	29.5 (16.2, 45.0)	7.0 (0, 18.8)
	*H*		2.249	1.389	2.113	1.025	2.545
	*P*		0.325	0.499	0.348	0.599	0.280
Swallow reaction	Normal	1	17	15	28	41	12
	Delayed	69	18.0 (4.0, 35.0)	12.0 (0, 27.0)	21.0 (11.0, 32.0)	35.0 (23.0, 49.0)	12.0 (0, 23.0)
	*H*		0.71	0.181	1.858	2.834	1.272
	*P*		0.4	0.671	0.173	0.092	0.259
Soft palate elevation	Normal	4	26.5 (17.5, 29.5)	23.5 (15.5, 29.0)	26.5 (20.5, 31.2)	36.5 (30.0, 43.5)	17.5 (14.2, 23.0)
	Impaired	33	16.0 (3.0, 30.0)	10.0 (0, 21.5)	20.0 (9.0, 29.0)	34.0 (19.5, 47.0)	12.0 (0, 21.0)
	Severe impaired	33	12.0 (2.0, 31.0)	7.0 (0, 24.0)	18.0 (9.0, 28.0)	30.0 (19.0, 46.0)	8.0 (0, 21.0)
	*H*		0.584	0.432	2.789	1.905	0.523
	*P*		0.56	0.651	0.069	0.157	0.595
Cricopharyngeal muscle opening	Intact	6	19.5 (16.2, 31.0)	15.5 (8.2, 23.8)	22.5 (11.2, 33.5)	34.5 (27.5, 44.5)	14.0 (9.2, 20.8)
	Inadequate	30	16.0 (4.8, 34.0)	10.0 (0, 24.5)	20.5 (9.8, 31.2)	34.5 (23.0, 47.2)	12.0 (0, 24.0)
	None	33	11.0 (1.0, 26.0)	6.0 (0, 16.0)	17.0 (7.5, 28.0)	29.0 (16.0, 44.0)	7.0 (0, 15.0)
	*H*		1.153	1.196	1.294	3.7	0.988
	*P*		0.322	0.309	0.281	0.030*	0.378
Glottic closure	Intact	14	21.0 (6.5, 39.2)	14.0 (2.8, 28.2)	22.5 (10.5, 34.5)	38.0 (25.0, 49.5)	14.0 (2.8, 23.0)
	Inadequate	38	14.5 (2.0, 30.5)	9.5 (0, 23.0)	18.5 (9.0, 30.2)	32.0 (18.8, 45.0)	9.5 (0, 21.2)
	None	19	14.0 (3.0, 30.0)	10.0 (0, 19.0)	21.0 (11.0, 32.0)	35.0 (22.0, 46.0)	12.0 (0, 21.0)
	*H*		0.263	0.805	0.203	0.877	1.011
	*P*		0.77	0.452	0.817	0.421	0.369
Vallecular & pyriform sinus residues	None	2	24.5 (21.8, 27.2)	15.0 (13.5, 16.5)	22.0 (18.0, 26.0)	35.0 (31.0, 39.0)	18.5 (13.2, 23.8)
	<10%	13	26.0 (11.0, 44.0)	15.0 (2.0, 32.0)	23.0 (11.5, 33.5)	41.0 (31.0, 54.0)	17.0 (8.0, 26.0)
	10–50%	37	15.0 (3.0, 30.0)	11.0 (0, 22.0)	20.0 (9.0, 30.0)	34.0 (21.0, 45.0)	9.0 (0, 20.0)
	>50%	18	6.0 (1.0, 18.5)	3.5 (0, 13.2)	12.0 (4.0, 23.5)	23.5 (12.8, 37.2)	4.0 (0, 14.0)
	*H*		1.391	1.672	1.378	3.811	1.026
	*P*		0.253	0.181	0.257	0.014*	0.387

^*^ *P* < 0.05 indicates significant difference in posterior Pmax among grades.

VFSS, Videofluoroscopic Swallowing Study.

No significant differences were observed in tongue pressure at any site across the FEES physiological components (all *P*>0.05, **Table 2**).

### Correlation with VFSS kinematic and temporal parameters

*Pearson* correlation showed that tongue Pmax at all five sites negatively correlated with oral transit time (*r* = -0.547 to -0.385, all *P<*0.001), with strongest associations for left and right anterior regions. Weak negative correlations were found between soft palate elevation time and Pmax at left anterior (*r* = -0.252, *P* = 0.036), middle (*r* = -0.248, *P* = 0.038), and right anterior (*r* = -0.248, *P* = 0.038). Hyoid movement time showed weak positive correlations with left anterior (*r* = 0.251, *P* = 0.036) and anterior (*r* = 0.270, *P* = 0.024) Pmax, and a moderate positive correlation with right anterior Pmax (*r* = 0.340, *P* = 0.011). Pharyngeal phase initiation time was negatively correlated with middle (*r* = -0.286, *P=*0.016) and posterior (*r=* - 0.313, *P=*0.008) Pmax. No significant correlations were observed between tongue Pmax and hyoid pause time, hyoid anterior movement (HAM), or hyoid superior movement (HSM) (all *P*>0.05).

### Association with aspiration on VFSS or FEES

Binary logistic regression showed that none of the five Pmax variables entered the regression equation for aspiration (PAS ≥ 6) on either VFSS (*χ²* =4.247, *df* =5, *P*= 0.514) or FEES (*χ²* =0.558, *df* =5, *P*= 0.990), indicating that tongue pressure at any site did not significantly predict aspiration on either assessment.

## Discussion

We used a T-shaped five-point sensor to compare region-specific maximal tongue pressure among patients with DSG, NDSG, and healthy controls, and analyzed correlations with swallowing function and aspiration. The multi-site assessment may also have methodological value. Single-site measurement can be influenced by sensor placement, individual tongue morphology, and habitual swallowing patterns, whereas a five-site profile provides a more comprehensive spatial representation of lingual pressure. This may improve the reliability of assessment and increase sensitivity for detecting region-specific impairment. In post-stroke patients, lingual weakness may not be uniform across sites; therefore, a single global value could obscure clinically relevant regional deficits. From a clinical perspective, site-specific pressure data may help inform individualized tongue-strength training, although this possibility requires prospective validation. A detailed discussion is provided as follows.

Across the three groups, the rank order of Pmax at the five test sites was generally consistent: the posterior tongue generated the highest pressure, whereas the right anterior tongue generated the lowest pressure. The left anterior and middle sites showed inter-individual variation in their relative order, and the anterior site was relatively low. A similar distribution has been reported in post-stroke populations (Hirota et al., 2010), and our findings suggest that this pattern may also be present in healthy individuals. The posterior tongue is biomechanically important for bolus propulsion (Rosen et al., 2017; Bahia and Lowell, 2024), which may partly explain the highest pressure at this site. Although previous studies have proposed possible neural mechanisms, including hemispheric contributions to anterior tongue control, fMRI activation patterns during swallowing (Martin et al., 2001), and compensatory mechanisms after stroke (Teismann et al., 2011), the present study did not assess neuroimaging findings, lesion side, handedness, or bolus placement. Therefore, these mechanisms cannot be confirmed in our cohort, and the cited findings should be regarded as background hypotheses rather than conclusions. Future studies combining fMRI, lesion-side information, handedness assessment, and kinematic measures are needed. Clinically, these findings provide site-specific reference data for tongue pressure measurement and may help inform individualized tongue-strength training.

In patients with post-stroke dysphagia, posterior Pmax differed significantly across groups with various degrees of cricopharyngeal opening. This finding aligns with Cock et al. (2017), supporting the role of posterior tongue pressure in upper esophageal sphincter (UES) opening. Although Konaka et al. (2010) did not directly quantify the relationship between posterior tongue pressure and UES opening, their study demonstrated that tongue pressure measurement can assess oral-phase pathophysiology in stroke patients, providing clinical support for our observation.

Posterior Pmax also varied significantly across groups with different severities of pharyngeal residue. Ono et al. (2007) noted that while tongue pressure facilitates bolus propulsion from the oral cavity to the pharynx, excessive propulsive force combined with inadequate pharyngeal clearance may paradoxically increase residue. Nakamori et al. (2024) confirmed a significant independent association between tongue pressure and pharyngeal residue in acute stroke patients. Pauloski et al. (2009) showed that insufficient tongue-base retraction and contact with the posterior pharyngeal wall is a key mechanism underlying pharyngeal residue. Thus, elevated tongue pressure accelerates bolus propulsion, but if pharyngeal clearance—mediated by pharyngeal wall contraction and laryngeal elevation—does not increase correspondingly, more residue may remain.

In contrast, no significant differences in Pmax at any of the five sites were observed across FEES-assessed physiological components. This negative finding is not unexpected but rather reflects inherent methodological differences between the two assessment tools. A systematic review reported that FEES is more sensitive than VFSS for detecting pharyngeal residue (Ferrari de Castro et al., 2025). Kelly et al. (2006) further found that FEES residue perception scores are systematically higher than those of VFSS, suggesting a lack of linear correspondence between FEES’s semi-quantitative scale and the continuous variable of tongue pressure. More importantly, Venkatesha et al (Venkatesha and Pawar, 2024). emphasized that VFSS is suitable for quantifying dynamic kinematic parameters of swallowing (e.g., temporal coupling between tongue pressure and cricopharyngeal opening), whereas FEES excels at evaluating pharyngeal structural morphology and airway protection. Langmore (2017) also noted that FEES cannot provide deep dynamic measurements. Consequently, although FEES may be preferred for assessing penetration/aspiration, its resolution for tongue-pharyngeal dynamics is limited, making it difficult to detect differences in tongue pressure across various pharyngeal physiological components.

Quantitative analysis of VFSS components revealed that all five Pmax values were negatively correlated with oral transit time, with the strongest correlations in the left and right anterior regions, indicating that anterior tongue pressure drives oral bolus transport efficiency. This is consistent with Lee et al. (2016), who found that both anterior and posterior hard palate tongue pressures correlate significantly with oral transit time, and that reduced tongue pressure prolongs swallowing timing parameters. Physiologic studies show that the anterior and middle tongue regions have the strongest voluntary control, generating higher pulsatile and clearing pressures, and greater applied pressure shortens oral half-emptying time (Pouderoux and Kahrilas, 1995). Positive correlations with hyoid movement time may represent a compensatory mechanism whereby higher tongue pressure enhances hyoid motion to assist pharyngeal swallowing. Pharyngeal phase initiation time was negatively correlated with middle and posterior Pmax, highlighting the posterior tongue’s role in triggering pharyngeal swallow.

In this study, neither VFSS nor FEES showed that Pmax significantly predicted aspiration risk. This negative finding statistically underscores the complexity of aspiration determination. Kojima et al. (2023) reported that tongue pressure in post-esophagectomy patients is only one of multiple risk factors for aspiration, not a sole determinant. Pizzorni et al. (2020) suggested that static maximal tongue pressure has limited discriminatory ability for aspiration risk, and additional parameters such as tongue endurance and neuromuscular reserve may be needed. A systematic review (Steele and Cichero, 2014) concluded that aspiration results from the interaction of multiple subsystems—respiration, tongue, hyoid, and larynx—and that measuring palatoglossal pressure alone, though valuable, cannot independently predict aspiration risk. Using the BOLUS framework, Palmer et al (Palmer and Padilla, 2024). further noted that aspiration-related adverse outcomes depend on several variables, including the viscosity and density of aspirated material, oral hygiene, and the patient’s overall condition. Therefore, we conclude that although reduced tongue pressure reflects diminished oral phase efficiency, aspiration involves multiple protective mechanisms—laryngeal elevation, glottic closure, cough reflex sensitivity, and pharyngeal sensation—thus a single maximal tongue pressure value is insufficient as a predictor of aspiration. The clinical value of maximal tongue pressure should therefore be confined to evaluating oral phase efficiency, rather than serving as an independent predictor of aspiration risk.

### Limitations

This study has several limitations. The validity and test–retest reliability of the five-point tongue pressure sensor require further validation. We did not compare additional tongue sites; therefore, the optimal number and location of measurement sites remain unclear. In addition, we did not assess neuroimaging findings, lesion side, handedness, or bolus placement. Consequently, the neural and behavioral mechanisms underlying the site-specific pressure distribution cannot be determined from the present data. Future multicenter, longitudinal studies combining neuroimaging, lesion-side information, handedness assessment, and kinematic measures are needed to validate the five-site approach and establish normative values.

Based on our findings, we recommend: (1) routine multi-point tongue pressure measurement in patients with post-stroke dysphagia; (2) use of left/right anterior Pmax to monitor oral-phase efficiency; (3) posterior tongue strengthening to improve pharyngeal function; (4) avoiding tongue pressure alone as an aspiration predictor—always combine with VFSS or FEES; (5) monitoring tongue function even in non-dysphagic stroke patients; and (6) developing individualized reference values accounting for age, sex, and stroke type.

## Conclusion

Five-point tongue pressure measurement objectively captures region-specific tongue weakness in post-stroke dysphagia, which correlates with prolonged oropharyngeal transit times. Tongue pressure has limited predictive value for aspiration and should be integrated into comprehensive swallowing assessment rather than used as a standalone screening tool.

## Data Availability

The original contributions presented in the study are included in the article/supplementary material. Further inquiries can be directed to the corresponding author.
